# TORC1 signaling regulates cytoplasmic pH through Sir2 in yeast

**DOI:** 10.1111/acel.13151

**Published:** 2020-05-25

**Authors:** Mayur Nimbadas Devare, Yeong Hyeock Kim, Joohye Jung, Woo Kyu Kang, Ki‐Sun Kwon, Jeong‐Yoon Kim

**Affiliations:** ^1^ Department of Microbiology and Molecular Biology College of Bioscience and Biotechnology Chungnam National University Daejeon Korea; ^2^ Aging Intervention Research Center Korea Research Institute of Bioscience and Biotechnology Daejeon Korea

**Keywords:** aging, cytoplasmic pH, Pma1, *Saccharomyces cerevisiae*, Sir2, TORC1

## Abstract

Glucose controls the phosphorylation of silent information regulator 2 (Sir2), a NAD^+^‐dependent protein deacetylase, which regulates the expression of the ATP‐dependent proton pump Pma1 and replicative lifespan (RLS) in yeast. TORC1 signaling, which is a central regulator of cell growth and lifespan, is regulated by glucose as well as nitrogen sources. In this study, we demonstrate that TORC1 signaling controls Sir2 phosphorylation through casein kinase 2 (CK2) to regulate *PMA1* expression and cytoplasmic pH (pHc) in yeast. Inhibition of TORC1 signaling by either *TOR1* deletion or rapamycin treatment decreased *PMA1* expression, pHc, and vacuolar pH, whereas activation of TORC1 signaling by expressing constitutively active *GTR1* (*GTR1*Q65L) resulted in the opposite phenotypes. Deletion of *SIR2* or expression of a phospho‐mutant form of *SIR2* increased *PMA1* expression, pHc, and vacuolar pH in the *tor1*Δ mutant, suggesting a functional interaction between Sir2 and TORC1 signaling. Furthermore, deletion of *TOR1* or *KNS1* encoding a LAMMER kinase decreased the phosphorylation level of Sir2, suggesting that TORC1 signaling controls Sir2 phosphorylation. It was also found that Sit4, a protein phosphatase 2A (PP2A)‐like phosphatase, and Kns1 are required for TORC1 signaling to regulate *PMA1* expression and that TORC1 signaling and the cyclic AMP (cAMP)/protein kinase A (PKA) pathway converge on CK2 to regulate *PMA1* expression through Sir2. Taken together, these findings suggest that TORC1 signaling regulates *PMA1* expression and pHc through the CK2–Sir2 axis, which is also controlled by cAMP/PKA signaling in yeast.

## Graphical abstract

TORC1 signaling and the cAMP/PKA pathway converge on CK2 to regulate PMA1 expression and cytoplasmic pH through Sir2. Cytoplasmic pH regulates TORC1 signaling and the cAMP/PKA pathway. Consequently, cytoplasmic pHc and TORC1 and cAMP/PKA signaling pathways form a kind of feedback loop to regulate cell growth in response to glucose availability.

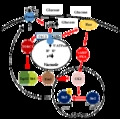

## 1 INTRODUCTION

Tor1 and Tor2 proteins in budding yeast are serine/threonine protein kinases belonging to the phosphoinositide 3‐kinases (PI3K)‐related kinase family (Keith & Schreiber, 1995). Rapamycin‐sensitive TORC1 connects nutrient availability and stress conditions to anabolic and catabolic activity to control cell growth (Loewith & Hall, 2011; Schmelzle & Hall, 2000). The limited supply of nutrients inhibits TORC1 activity, which leads to the extension of lifespan in yeast as well as in other various organisms (Bjedov et al., 2010; Harrison et al., 2009; Kaeberlein et al., 2005). In yeast, TORC1 activity is regulated through the vacuolar membrane‐associated EGO complex, (Gao & Kaiser, 2006), in which Gtr1 and Gtr2 are Ras‐family GTPases and orthologs of the metazoan counterparts RagA/B and Rag C/D, respectively (Kim, Goraksha‐Hicks, Li, Neufeld, & Guan, 2008; Sancak et al., 2008). Gtr1 loaded with GTP by the guanine nucleotide exchange factor Vam6 interacts with and activates TORC1 depending on amino acid availability, leucine and glutamine in particular (Binda et al., 2009; Powis & De Virgilio, 2016). Interestingly, Gtr1/2 GTPases also mediate glucose signaling to TORC1 (R. Dechant, Saad, Ibanez, & Peter, 2014; Efeyan et al., 2013).

Activation of Sir2, a NAD^+^‐dependent deacetylase, plays an important role in lifespan extension in yeast, worms, flies, and mice (Kaeberlein, McVey, & Guarente, 1999; Rogina & Helfand, 2004; Satoh et al., 2013; Tissenbaum & Guarente, 2001). Sir2 extends lifespan by inhibiting the formation of extrachromosomal rDNA circles (ERCs) in yeast (Huang & Moazed, 2003; Kaeberlein et al., 1999); it has also been reported that Sir2 regulates replicative lifespan (RLS) through deacetylating histone H4 lysine 16 (H4K16) at the subtelomeric regions (Dang et al., 2009). Recently, our group proposed a novel mechanism for Sir2 role in extending RLS: Sir2 phosphorylated by the activated PKA–casein kinase 2 (CK2) axis derepresses *PMA1* transcription and decreases lifespan (Kang, Kim, Kang, Kwon, & Kim, 2015).

Pma1 is a P‐type H^+^‐ATPase that transports protons across the plasma membrane and functions as a major regulator of cytoplasmic pH (pHc) in yeast (Orij, Brul, & Smits, 2011; Serrano, Kielland‐Brandt, & Fink, 1986). As pHc tightly correlates with cell growth (R. Dechant et al., 2014), understanding the regulatory mechanisms of *PMA1* expression and activity is crucial to know how yeast cells respond to a variety of nutritional and environmental factors, such as glucose and acidic pH, to maintain cellular processes for growth and survival. Pma1 is reversibly activated by phosphorylation at the C‐terminal tail in response to glucose (Chang & Slayman, 1991; Eraso & Portillo, 1994; Serrano, 1983) and also transcriptionally regulated by glucose (Portillo, 2000). Interestingly, it has been recently reported that inhibition of TORC1 signaling reduces Pma1 activity almost by half, although detailed mechanisms remain elusive (Deprez, Eskes, Wilms, Ludovico, & Winderickx, 2018). However, the role of TORC1 in Pma1 activity regulation does not seem to be affected by N sources or amino acids, because addition of various amino acids does not alter pHc (Dechant et al., 2014; Wilms et al., 2017). Thus, it is likely that regulation of Pma1 expression and activity for pH homeostasis in yeast would be largely dependent on glucose availability.

Sir2 receives a signal for Pma1 expression regulation from the cAMP/PKA–CK2 axis activated by glucose (Kang et al., 2015), and TORC1 signaling is also regulated by glucose (Prouteau et al., 2017). Sir2, cAMP/PKA signaling, and TORC1 signaling are major players in determining longevity in response to nutrient availability (Dilova, Easlon, & Lin, 2007). Furthermore, TOR signaling negatively regulates Sir2 function toward ERC formation to extend lifespan in yeast (Ha & Huh, 2011; Medvedik, Lamming, Kim, & Sinclair, 2007), and a similar negative relationship between sirtuins and mTOR has been also observed in mammals (Ghosh, McBurney, & Robbins, 2010; Guo et al., 2011). Based on these reports, we hypothesized that Sir2 and TORC1 signaling may be somehow connected to each other to regulate Pma1 expression. Here, we demonstrate that TORC1 signaling regulates Pma1 expression to control pHc, and vacuolar acidity through Sir2. Additionally, we proved that TORC1 and cAMP/PKA signaling pathways converge on CK2 kinase to regulate Sir2.

## 2 RESULTS

### 2.1 TORC1 signaling regulates *PMA1* expression through Sir2

**Figure 1:**
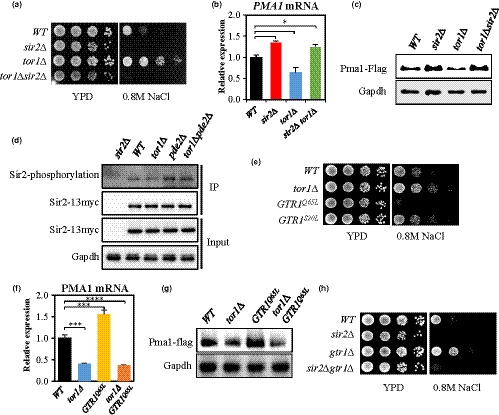
Change in TORC1 activity modulates *PMA1* expression through Sir2. (a) Salt stress resistance was tested by spotting the wild‐type (BY4741), *sir2*Δ, *tor1*Δ, and *sir2*Δ *tor1*Δ strains on YPD with or without 0.8 M NaCl and incubating the plates for three days. (b) *PMA1* mRNA levels were measured in the indicated strains by qRT–PCR (*p < .05). (c) Pma1‐FLAG tag was expressed chromosomally and analyzed by Western blot in the indicated strains. (d) Sir2 phosphorylation level in *WT*, *tor1*Δ, *pde2*Δ, and *pde2*Δ *tor1*Δ strains. Sir2‐myc13 expressing strains were immunoprecipitated (IP) with anti‐myc antibody and analyzed by Western blotting as indicated. *Sir2∆* strain was used as a negative control. (e) Salt stress resistance was tested by spotting the *WT*, *tor1*Δ, *GTR1^Q65L^*, and *GTR1^S20L^* strains on YPD with or without 0.8 M NaCl and incubating the plates for three days. (f) *PMA1* mRNA levels were measured in the indicated strains by qRT–PCR (***p < .001, ****p < .0001). (g) Pma1 protein levels were analyzed by Western blot in the indicated strains. GAPDH was used as the loading control. (h) Salt stress resistance was tested by spotting the *WT*, *sir2*Δ, *gtr1*Δ, and *sir2*Δ *gtr1*Δ strains on YPD with or without 0.8 M NaCl and incubating the plates for three days. Values in (b) and (f) represent average of at least three independent experiments (±*SD*)

A previous report has demonstrated that inhibition of TORC1 signaling stabilizes rDNA loci by modulating the association of Sir2 with rDNA region, indicating a cross‐talk between TORC1 signaling and Sir2 activity (Ha & Huh, 2011). We were curious about whether TORC1 signaling is also associated with Sir2 activity to regulate *PMA1* expression in *Saccharomyces cerevisiae*. To investigate the possibility, NaCl stress resistance was exploited as a phenotypic readout because *sir2*Δ cells are sensitive to NaCl due to increased *PMA1* expression (Kang et al., 2015). The *tor1*Δ mutant was highly resistant to NaCl compared to the wild‐type strain (Figure 1a). Interestingly, the NaCl‐resistant phenotype of *tor1*Δ was completely eradicated by *SIR2* deletion (Figure 1a), suggesting a possibility that TORC1 signaling controls NaCl resistance through Sir2. *PMA1* expression levels were measured in the *tor1*Δ and *tor1*Δ *sir2*Δ mutant strains because the transcriptional repression of *PMA1* by Sir2 contributes to NaCl resistance (Kang et al., 2015). The amount of *PMA1* mRNA was lower in the *tor1*Δ strain than that in the wild‐type (Figure 1b), which is consistent with the observation that treatment of rapamycin, an inhibitor of TORC1, decreased *PMA1* mRNA level (Figure S1a). However, *SIR2* deletion in the *tor1*Δ strain elevated *PMA1* expression level up to that observed in the *sir2*Δ strain (Figure 1b). In addition, the differences in *PMA1* mRNA level were similarly reflected in the amount of Pma1 protein (Figure 1c). The involvement of Tor1 in the regulation of *PMA1* expression was valid in another strain (Figures S1b,c), suggesting that TORC1 signaling may generally control Sir2 activity to regulate *PMA1* expression in *S. cerevisiae*. Next, we measured the phosphorylation status of Sir2 in *tor1*Δ and wild‐type strains and found that lack of Tor1 decreased the phosphorylation level of Sir2 (Figure 1d), which indicates that TORC1 signaling is involved in the Sir2 phosphorylation that controls *PMA1* expression (Kang et al., 2015).

Glucose regulates Sir2 activity through cAMP/PKA (Kang et al., 2015) and activates TORC1 signaling through the Rag GTPases Gtr1/Gtr2 (Dechant et al., 2014). Hence, we determined whether Gtr1 is involved in the activation of TORC1 signaling to regulate *PMA1* expression through Sir2. To answer the question, we examined the NaCl stress resistance of cells expressing *GTR1^Q65L^* or *GTR1^S20L^* allele, which is restricted to an active GTP‐bound or inactive GDP‐bound confirmation, respectively (Binda et al., 2009; Nakashima, Noguchi, & Nishimoto, 1999). Constitutive activation of Gtr1 (*GTR1^Q65L^*) resulted in a hypersensitive phenotype to NaCl, whereas inactivation of Gtr1 (*GTR1^S20L^*) increased resistance to NaCl stress (Figure 1e). mRNA and protein levels of Pma1 were higher in *GTR1^Q65L^*‐expressing cells than those in the wild‐type but were similarly low in the *tor1*Δ and *tor1*Δ *GTR1^Q65L^* strains (Figure 1f,g). Moreover, the NaCl‐resistant phenotype of the *gtr1∆* strain was abolished in the *gtr1∆ sir2∆* strain (Figure 1h). We also examined whether Pib2 participates in *PMA1* expression regulation because Pib2 is required to reactivate TORC1 following rapamycin‐induced growth arrest, independently of the Rag GTPases Gtr1/Gtr2 (Ukai et al., 2018; Varlakhanova, Mihalevic, Bernstein, & Ford, 2017). However, the minor effect of *PIB2* deletion on *PMA1* mRNA and protein levels (Figure S2) suggested that Pib2 is not involved in the activation of TORC1 signaling for *PMA1* expression regulation. Collectively, these results led us to conclude that Gtr1/2 GTPases control TORC1 signaling to regulate *PMA1* expression through Sir2.

### 2.2 TORC1 signaling controls pHc and vacuolar pH

**Figure 2:**
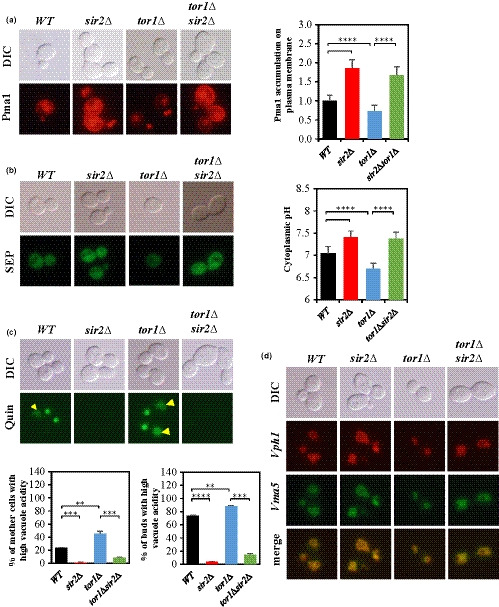
TORC1 signaling regulates the amount of Pma1 and pHc and vacuolar pH. (a) In exponentially growing cells, the amount of Pma1‐mCherry localized at the plasma membrane was determined by measuring the fluorescence intensity in the *WT*, *sir2*Δ, *tor1*Δ, and *sir2*Δ *tor1*Δ strains. (b) pHc was determined by measuring fluorescence intensity of pH‐sensitive SEP. (c) Vacuolar acidity was determined as indicated by quinacrine staining of exponentially growing cells. (d) V‐ATPase assembly in exponentially growing cell was analyzed by fluorescence microscopy by co‐expressing Vph1‐mCherry (a component of the Vo sector) and Vma5‐GFP (a component associated with the V_1_ sector). (a), (b), and (c), *n* > 100 cells for all strains. A representative image is shown for each strain

pHc and vacuolar pH in yeast are primarily controlled by Pma1 (Reinhard Dechant et al., 2010) and the vacuolar H^+^‐ ATPase (V‐ATPase) (Forgac, 2007; Kane, 2006), respectively. As Sir2 and TORC1 signaling are associated with *PMA1* expression, we hypothesized that pHc of the *sir2*Δ or *tor1*Δ mutant cells would be different from that of the wild‐type. To test the hypothesis, we measured the amount of Pma1 localized to the plasma membrane and pHc of the *sir2*Δ, *tor1*Δ, and *sir2*Δ *tor1*Δ cells growing exponentially in a glucose‐rich complex medium. Pma1 tagged with mCherry was expressed from its native promoter, and the pH‐sensitive super‐ecliptic pHluorin (SEP) (Sankaranarayanan, De Angelis, Rothman, & Ryan, 2000) was expressed from the *TEF1* promoter to measure pHc. Although the amount of Pma1 localized to the plasma membrane increased in the *sir2∆* mutant compared to that in the wild‐type strain, less amount of Pma1 was present on the plasma membrane in the *tor1∆* strain than that in the wild‐type (Figure 2a). Moreover, the deletion of *SIR2* in the *tor1∆* mutant increased Pma1 present on the plasma membrane up to the level of that observed in the *sir2∆* mutant (Figure 2a). The change in the amount of Pma1 on the plasma membrane was correlated with pHc; pHc was higher in the *sir2*Δ mutant and lower in the *tor1∆* mutant than that in the wild‐type, and pHc was similar in the *sir2*Δ *tor1*Δ and *sir2*Δ mutants (Figure 2b). These results suggest that the regulation of *PMA1* expression by TORC1 signaling through Sir2 plays an important role in maintaining the pHc of yeast cells growing exponentially in a glucose‐rich complex medium.

Next, we measured vacuolar acidity in the *sir2*Δ, *tor1*Δ, and *sir2*Δ *tor1*Δ mutant strains because an increased accumulation of Pma1 on the plasma membrane of aged cells negatively affects vacuolar acidity (Henderson, Hughes, & Gottschling, 2014). The portions of the mother and newly budding cells with high vacuolar acidity decreased significantly in the *sir2∆* mutant and increased in the *tor1∆* mutant compared to those in the wild‐type (Figure 2c). Again, *SIR2* deletion in the *tor1∆* mutant significantly reduced the portions of cells with high vacuolar acidity (Figure 2c). Because we found similar phenotypes in vacuolar acidity of the mutant strains with cells purified at five divisions (Figure S4a), we continued to experiment without purifying cells at a specific age. V‐ATPase activity is essential for vacuole acidification in response to glucose metabolism and is regulated by the assembly of V‐ATPase subunits on the vacuolar membrane (Martinez‐Munoz & Kane, 2008; Wilms et al., 2017). Thus, we examined whether the *sir2∆*, *tor1∆*, and *sir2∆ tor1∆* mutant strains differed in V‐ATPase assembly. V‐ATPases appeared normally assembled in the strains proliferating exponentially in a glucose‐rich medium and disassembled in glucose‐starved medium (Figure 2d and FigureS4b), indicating no difference in V‐ATPase assembly in the mutant strains. These results suggest that the regulation of *PMA1* expression by TORC1 signaling through Sir2 affects vacuolar acidification independently of V‐ATPase activation.

### 2.3 TORC1 signaling regulates *PMA1* expression through Sir2 phosphorylation at the serine 473 residue

**Figure 3:**
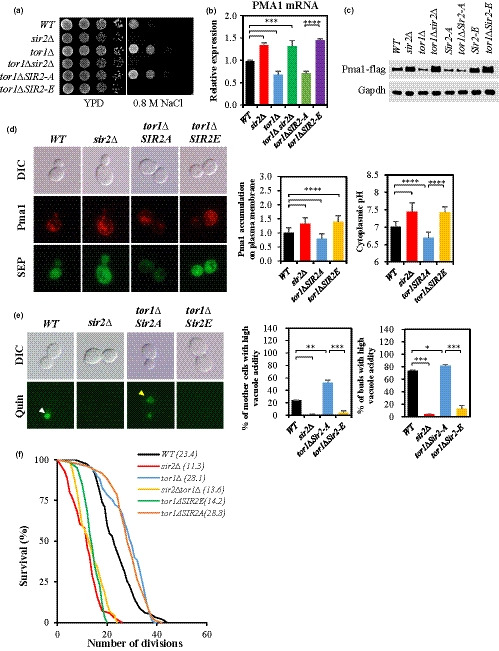
TORC1 signaling regulates *PMA1* expression through Sir2 phosphorylation at the S473 residue. (a) Salt stress resistance was tested by spotting the *WT*, *sir2*Δ, *tor1*Δ, *sir2*Δ *tor1*Δ, *tor1*Δ *SIR2‐A*, and *tor1*Δ *SIR2‐E* strains on YPD with or without 0.8 M NaCl and incubating the plates for three days. (b) *PMA1* mRNA levels were measured in the indicated strains by qRT–PCR. (c) Pma1‐FLAG tag was expressed chromosomally and analyzed by Western blot in the indicated strains. (d) In exponentially growing cells, Pma1 accumulation and pHc were determined by measuring the fluorescence intensity. (e) Vacuolar acidity was determined as indicated by quinacrine staining of exponentially growing cells. (f) RLS of the *WT*, *sir2*Δ, *tor1*Δ, *sir2*Δ *tor1*Δ, *tor1∆SIR2‐A*, and *tor1∆SIR2‐E* strains. *p* < .0001 (*WT* versus. *sir2*Δ), *p* < .0001 (*WT* versus. *tor1*Δ), *p* < .0001 (*WT* versus. *tor1*Δ *sir2*Δ), and *p* < .0001 (*tor1∆ SIR2‐A* versus*. tor1∆ SIR2‐E).* *n* = 72 for all strains. RLS was measured by micromanipulation. The mean lifespan is indicated. (d) and (e), *n* > 100 cells for all strains. A representative image is shown for each strain. Values in (b) represent average of at least three independent experiments (±*SD*)

Our previous study revealed that the phosphorylation of Sir2 at the serine 473 (S473) residue, a conserved phosphorylation site in sirtuin family proteins, is essential for Sir2 to repress *PMA1* expression (Kang et al., 2015) and this study uncovered that deletion of *TOR1* decreased the phosphorylation level of Sir2 (Figure 1d). Thus, we examined whether TORC1 signaling regulates *PMA1* expression through Sir2 phosphorylation at S473. Expression of a phospho‐mimetic form of Sir2 (*SIR2^S473E^*) in the *tor1*Δ strain abrogated NaCl stress resistance and increased *PMA1* mRNA and protein levels as high as those observed in the *sir2∆* or *tor1*Δ *sir2*Δ strains (Figure 3a–c), indicating the importance of Sir2 phosphorylation at S473 for the regulation of *PMA1* expression by TORC1 signaling. We also determined the involvement of Sir2 phosphorylation in the regulation of pHc by TORC1 signaling. pHc was elevated in the *tor1∆* strain expressing *SIR2^S473E^*, similar to that observed in the *sir2*Δ strain, but decreased in the *tor1∆* strain expressing a phospho‐defective form (*SIR2^S473A^*) (Figure 3d). In addition, the increase in vacuolar acidity by *TOR1* deletion decreased significantly by *SIR2^S473E^* expression, but the expression of *SIR2^S473A^* did not cause a noticeable change in vacuolar acidity in the *tor1*Δ mutant (Figures 2c and 3e). Further, we investigated whether inhibition of TORC1 signaling affects RLS through Sir2 phosphorylation at S473. Expression of the phospho‐mimetic *SIR2^S473E^* decreased RLSs of the *tor1∆* mutant strain, whereas expression of a phospho‐defective *SIR2^S473A^* had little effect on RLS of the *tor1∆* mutant (Figure 3f). These findings support that TORC1 signaling contributes to the regulation of *PMA1* expression and pHc through Sir2 phosphorylation at the S473 residue that is associated with the regulation of RLS in yeast.

### 2.4 Kns1 acts downstream of TORC1 signaling to regulate *PMA1* expression

**Figure 4:**
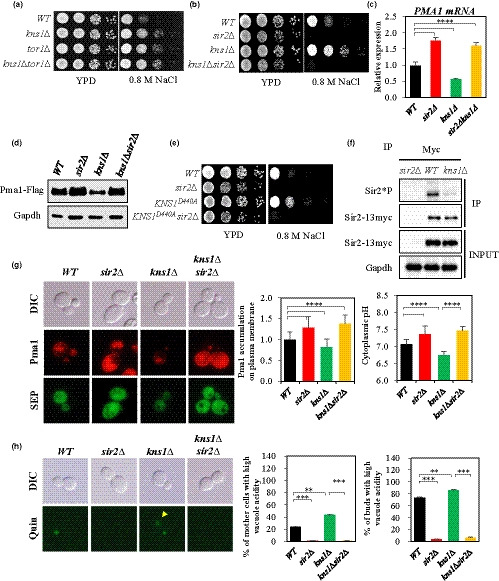
Kns1 acts downstream of TORC1 to regulate *PMA1* expression. (a), (b), and (e) Salt stress resistance was analyzed by spotting the *WT*, *kns1*Δ, *tor1*Δ, and *kns1* Δ *tor1*Δ (a); *WT*, *sir2*Δ, *kns1*Δ, and *kns1*Δ *sir2*Δ (b); and *WT*, *sir2*Δ, *KNS1^D440A^*, and *KNS1^D440A^ sir2*Δ (e) strains on YPD with or without 0.8 M NaCl and incubating the plates for three days. (c) *PMA1* mRNA levels were measured in the indicated strains by qRT–PCR (****p < .0001). (d) Pma1‐FLAG tag was expressed chromosomally and analyzed by Western blot. (f) Sir2 phosphorylation level in *WT* and *kns1*Δ strains. Sir2‐myc13 expressing strains were immunoprecipitated (IP) with anti‐myc antibody and analyzed by Western blotting as indicated. *Sir2∆* strain was used as a negative control. (g) In exponentially growing cells, Pma1 accumulation and pHc were determined by measuring the fluorescence intensity. (h) Vacuolar acidity was determined as indicated by quinacrine staining of exponentially growing cells. (g) and (h), *n* > 100 cells for all strains. A representative image is shown for each strain. Values in (c) represent average of at least three independent experiments (±*SD*)

Kns1, a LAMMER kinase, is regulated by TORC1 signaling and mediates the phosphorylation of the RNA polymerase subunit Rpc53 and a CK2 subunit in response to nutrient limitation or various cellular stresses (Lee, Moir, McIntosh, & Willis, 2012; Sanchez‐Casalongue et al., 2015). Thus, we determined whether Kns1 is involved in TORC1 signaling for the regulation of *PMA1* expression and pHc. It was found that cells deleted for *KNS1* were highly resistant to NaCl, similar to that observed in the *tor1∆* strain, and there was no additive effect of simultaneous deletion of *KNS1* and *TOR1* on NaCl resistance (Figure 4a). In addition, *SIR2* deletion in the *kns1∆* mutant highly reduced the resistance to NaCl and increased the amount of *PMA1* mRNA and protein (Figure 4b,d), and expression of a kinetically inactive *KNS1* (*KNS1* ^D440A^) (Lee et al., 2012) imparted NaCl resistance to the wild‐type strain (Figure 4e). Furthermore, we found that lack of Kns1 significantly decreased the phosphorylation level of Sir2 (Figure 4f), supporting that Kns1 plays a role in regulating *PMA1* expression through Sir2 phosphorylation.

Subsequently, we assessed whether Kns1 is required for maintaining pHc and vacuolar acidity. The *kns1∆* mutant showed a decreased amount of Pma1 localized to the plasma membrane and low pHc compared to those observed in the wild‐type strain (Figure 4g), and deletion of *SIR2* in the *kns1∆* mutant increased the amount of Pma1 present on the plasma membrane and pHc up to the levels observed in the *sir2∆* mutant (Figure 4g). In addition, the portion of mother cells with high vacuolar acidity increased in the *kns1∆* mutant and significantly decreased in the *kns1∆ sir2∆* mutant compared to that in the wild‐type (Figure 4h). We further tested whether expression of *SIR2^S473E^* altered the phenotypes of the *kns1*Δ mutant. The *kns1*Δ mutant expressing *SIR2^S473E^* was more sensitive to NaCl and produced less acidic vacuoles than those by the wild‐type and *kns1∆* strains (Figure S5). Collectively, these results suggest that Kns1, as a part of TORC1 signaling, is involved in *PMA1* expression regulation through Sir2 phosphorylation at S473.

### 2.5 Sit4 phosphatase mediates TORC1 signaling for *PMA1* expression regulation

**Figure 5:**
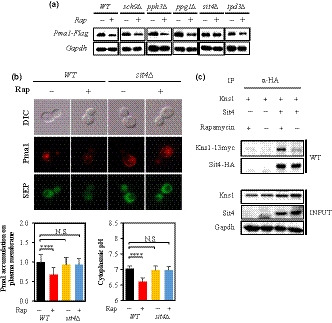
Sit4 phosphatase is involved in TORC1 signaling for *PMA1* expression regulation. (a) The *sch9*Δ, *pph3*Δ, *ppg1*Δ, *sit4*Δ, and *tpd3*Δ mutant strains were grown exponentially and then treated or not (mock) with 300 nM rapamycin for 1 hr, and Pma1 protein level was analyzed by Western blot. (b) Exponentially growing *WT* and *sit4∆* cells were treated or not with rapamycin as in (a), and Pma1 accumulation and pHc were determined by measuring the fluorescence intensity. *n* > 100 cells for all strains. A representative image is shown for each strain. (c) Cells co‐expressing *KNS1*‐myc13 and *SIT4*‐HA were grown exponentially in YPD and treated or not (mock) with 300 nM rapamycin for 1 hr, and extracts were immunoprecipitated with anti‐HA antibody. Whole‐cell extract and immunoprecipitated proteins were analyzed by Western blotting

TORC1 signaling controls cellular growth primarily through two main downstream effectors, the AGC kinase Sch9 and Tap42‐regulated phosphatase complex, that are directly phosphorylated by TORC1 (Jacinto, Guo, Arndt, Schmelzle, & Hall, 2001; Jiang & Broach, 1999). First, we examined whether Sch9 is involved in the regulation of *PMA1* expression by TORC1 signaling. *PMA1* expression levels of the wild‐type and *sch9∆* cells were similar when no rapamycin treatment was administered (Figure S6). However, the Pma1 protein level in *sch9∆* cells decreased upon rapamycin treatment as was the case in the wild‐type (Figure 5a). Thus, we concluded that Sch9 is not associated with TORC1 signaling for the regulation of *PMA1* expression. Then, we examined whether TORC1 signaling regulates *PMA1* expression through Tap42‐regulated phosphatases. Mutants deleted for individual catalytic phosphatase subunits (*PP4/pph3∆*, *PPG1/ppg1∆*, and *PP6/sit4∆*) of PP2A‐like protein phosphatases or the common regulatory subunit (*tpd3∆*) of PP2A phosphatases (Hombauer et al., 2007; Workman, Chen, & Laribee, 2016) were generated and examined for the change in Pma1 protein level by rapamycin treatment. Rapamycin treatment reduced Pma1 protein level in the wild‐type and all phosphatase mutants, except for in the *sit4∆* cells (Figure 5a), suggesting that the effect of TORC1 inhibition on *PMA1* expression is achieved through Sit4. To further investigate the involvement of Sit4 in the regulation of *PMA1* expression by TORC1 signaling, we measured the amount of Pma1 on the plasma membrane and pHc before and after rapamycin treatment in the *sit4∆* and wild‐type cells. Rapamycin treatment reduced the amount of Pma1 on the plasma membrane and lowered pHc in the wild‐type, but not in the *sit4∆* strain (Figure 5b), which supports that Sit4 is required for the regulation of *PMA1* expression by TORC1 signaling. As both Sit4 and Kns1 participate in TORC1 signaling to regulate *PMA1* expression, a possibility is that Sit4 and Kns1 may form an epistatic relationship in the signaling pathway. Co‐immunoprecipitation experiment revealed that Sit4 and Kns1 physically interact with each other in a rapamycin‐dependent manner (Figure 5c), suggesting that Sit4 released from Tap42 upon rapamycin treatment may control Kns1 activity because Sit4 is a direct downstream effector of TORC1. Taken together, these results suggest that TORC1 signaling regulates *PMA1* expression through Sit4, probably via Sit4–Kns1.

### 2.6 TORC1 and cAMP/PKA signaling pathways converge on CK2 to regulate *PMA1* expression

**Figure 6:**
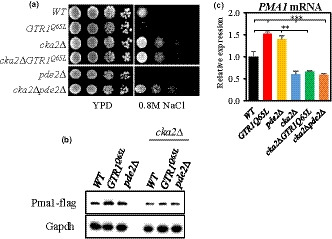
TORC1 signaling regulates *PMA1* expression through CK2. (a) Salt stress resistance was analyzed by spotting the *WT*, *GTR1^Q65L^*, *cka2*Δ, *cka2*Δ *GTR1^Q65L^*, *pde2*Δ, and *cka2*Δ *pde2*Δ strains on YPD with or without 0.8 M NaCl and incubating the plates for three days. (b) *PMA1* mRNA levels were measured in the indicated strains by qRT–PCR. **p < .01 (*WT* versus. *pde2∆* and *cka2∆ GTR1^Q65L^*), *** *p* < .001 (*WT* versus. *GTR1^Q65L^*, *cka2∆*, and *cka2∆ pde2∆*). (c) Pma1‐FLAG was expressed chromosomally in the indicated strains and analyzed by Western blot

The cAMP/PKA signaling pathway regulates *PMA1* expression through the CK2–Sir2 axis (Kang et al., 2015). Thus, our finding that TORC1 signaling regulates *PMA1* expression through Sir2 prompted us to hypothesize that TORC1 signaling may also regulate *PMA1* expression via the CK2–Sir2 axis. To test the hypothesis, *CKA2* encoding a catalytic subunit of CK2 was deleted from the strain expressing a TORC1‐activating *GTR1^Q65L^* allele and checked for sensitivity to NaCl. The *GTR1^Q65L^* strain became resistant to NaCl in the absence of Cka2, which was similar to the phenotypic change of the *pde2∆* mutant by *CKA2* deletion (Figure 6a). Moreover, *CKA2* deletion reduced *PMA1* mRNA and protein levels in the *GTR1^Q65L^* and *pde2∆* mutants as well as in the wild‐type (Figure 6b,c). These results suggest that TORC1 signaling is connected to Sir2–Pma1 via CK2 as with the cAMP/PKA signaling pathway. Furthermore, we observed that the introduction of *PDE2* deletion into the *tor1∆* mutant resulted in an intermediate NaCl‐resistant phenotype and *PMA1* mRNA level compared to those in the *pde2∆* and *tor1∆* mutants (Figure S7). Taken together, these results suggest that TORC1 signaling and the cAMP/PKA pathway converge on CK2 to regulate *PMA1* expression through Sir2.

## 3 DISCUSSION

TORC1, which is a highly conserved protein kinase complex from yeast to humans, controls cell growth by regulating ribosome biogenesis, protein synthesis, autophagy, and entry into the reversible quiescent state/Go (Loewith & Hall, 2011). In this study, we showed that TORC1 signaling is involved in the regulation of *PMA1* expression, pHc, and vacuolar pH. We also uncovered that TORC1 signal is delivered to CK2 through Sit4 and affects Sir2 phosphorylation at the S473 residue, which in turn controls *PMA1* expression. Together with our previous results demonstrating that cAMP/PKA signaling regulates *PMA1* expression and RLS through the CK2–Sir2 axis, this study provides us with very intriguing information that two of the most important nutrient signaling pathways, TORC1 and cAMP/PKA, converge on CK2 to regulate Sir2 activity toward *PMA1* expression, pHc, and vacuolar pH in *S. cerevisiae*.

TORC1 and PKA signaling pathways coordinate cellular processes for growth in response to nutrients and stress conditions in yeast. The two pathways converge on the protein kinase Rim15 to properly control the entry into a quiescent state (Pedruzzi et al., 2003) and regulate a few common target proteins that are important for growth and stress responses (Beck & Hall, 1999; Gorner et al., 1998; Jorgensen et al., 2004), although whether they function dependently or interact with each other for the purpose is not determined unequivocally. According to one model, TORC1 and PKA signaling pathways function in parallel to promote cell growth or control autophagy (Ramachandran, Shah, & Herman, 2011; Stephan, Yeh, Ramachandran, Deminoff, & Herman, 2009), but the other model proposes that TORC1 is upstream of the PKA pathway (Martin, Demougin, Hall, & Bellis, 2004; Schmelzle, Beck, Martin, & Hall, 2004) and activates only a subset of PKA substrates (Soulard et al., 2010). In this study, we report that the TORC1 and cAMP/PKA signaling pathways converge on the protein kinase CK2 to control *PMA1* expression through Sir2. Activation or inactivation of one of the pathways increased or decreased *PMA1* expression, respectively, when the other was normally functioning, but the effect of activation of cAMP/PKA signaling on *PMA1* expression was compromised by inactivation of TORC1 signaling (Figure S7). Thus, it is likely that two signals from TORC1 and PKA combine together to control the intensity of the overall signal for regulating pHc and maintaining proper growth according to environmental conditions, although we do not know the detailed mechanism of how each of the two pathways regulates CK2 activity.

A recent study reported that inhibition of TORC1 signaling reduces Pma1 activity to 57% of the control cells (Mahmoud et al., 2017). As Pma1 activity was measured from the total membrane fraction, not from purified Pma1, in the study, the reduced Pma1 activity is likely to be due to its downregulated expression level. If this were the case, the result would be consistent with our data showing that inhibition of TORC1 signaling downregulates *PMA1* expression. The study also reported that the deletion of *SIT4* decreases Pma1 activity and lowers pHc compared to that in the wild‐type (Mahmoud et al., 2017). In this study, however, we found that the deletion of *SIT4* did not alter the levels of Pma1 and pHc (Figure 5b). If Sit4 detached from Tap42 upon TORC1 inactivation is involved in *PMA1* expression regulation, the absence of Sit4 is expected to alter *PMA1* expression. However, if most Sit4 proteins are bound to Tap42 and are maintained as inactivated in glucose‐rich condition wherein TORC1 is active (Di Como & Arndt, 1996), there may be little difference, if any, in Pma1 levels between the *sit4∆* mutant and wild‐type cells growing in glucose‐rich condition, which is in agreement with our data (Figure 5b). Further studies are needed to explain the reason for the discrepancy between the results of this study and the previous publication (Mahmoud et al., 2017).

Kns1 is the only member of the LAMMER/CDC‐like (CLK) kinase family in yeast. A number of studies in different organisms have revealed a function of LAMMER kinases to phosphorylate serine/arginine‐rich (SR) proteins that regulate mRNA splicing (Colwill et al., 1996; Du, McGuffin, Dauwalder, Rabinow, & Mattox, 1998; Nikolakaki et al., 2002). However, less is known about its function in other processes. Recent studies have reported that Kns1 is a downstream effector of TORC1 in the regulation of ribosome and tRNA synthesis and functions by priming the phosphorylation of Rpc53 by Mck1 (Lee et al., 2012); Kns1 directly phosphorylates Cbk1, a regulatory subunit of CK2, to regulate the association of CK2 with some of its substrates (Sanchez‐Casalongue et al., 2015). In this study, we discovered that Kns1 is positively controlled by TORC1 to regulate Sir2 activity and *PMA1* expression. In addition, because we detected a direct interaction between Sit4 and Kns1 (Figure 5c), we speculate that Sit4 freed from Tap42 when TORC1 signaling is inhibited might inhibit Kns1 through dephosphorylation, which in turn inhibit CK2 from phosphorylating Sir2. Further study is expected to uncover how Sit4 is related to Kns1 to control *PMA1* expression regulation.

Alteration in pHc causes an enormous impact on cell growth as it could change enzyme activity, protein structure, membrane potential, functions of subcellular organelles, etc. (Deprez et al., 2018; Orij et al., 2011). Because Pma1 effluxes protons from the cytoplasm to the extracellular space to control pHc (Morsomme, Slayman, & Goffeau, 2000), tight regulation of activity and expression level of Pma1 in accordance with the environmental condition would be crucial for maintaining optimal cell growth and increasing survival and longevity. In addition, low pHc due to the asymmetric distribution of Pma1 on the plasma membrane is responsible for the rejuvenation of daughter cell budding from old mother cells (Henderson et al., 2014). Glucose activates Pma1 post‐translationally by inducing phosphorylation at serine 911 and threonine 912 residues of Pma1 (Lecchi et al., 2007; Mazon, Eraso, & Portillo, 2015) and increases the transcription of *PMA1* by controlling Sir2 phosphorylation and binding of the Rap1 transcription factor to the upstream activating sequence of *PMA1* (Capieaux, Vignais, Sentenac, & Goffeau, 1989; Rao, Drummond‐Barbosa, & Slayman, 1993). Thus, in glucose‐rich condition, wherein Pma1 is fully active and the *PMA1* promoter is highly activated, it is possible that V‐ATPase may contribute to pHc regulation. V‐ATPase, which is a multi‐subunit enzyme structurally organized into two major sectors, a peripheral V1 sector for ATP hydrolysis and a membrane‐embedded Vo sector for proton translocation (Parra, Chan, & Chen, 2014), is a primary proton pump localized on the vacuolar membrane and is responsible for the glucose‐mediated increase in vacuolar acidity (Orij et al., 2011). Sch9 kinase, a major downstream effector of TORC1, is involved in the regulation of V‐ATPase by affecting reversible disassembly of the V_o_ and V_1_ sectors in response to glucose availability (Kane, 1995; Parra et al., 2014; Wilms et al., 2017). However, we found that Sch9 has no role in *PMA1* expression regulation by TORC1 signaling in glucose‐rich condition (Figure S6), and V‐ATPase remained assembled in the *sir2∆* and *tor1∆* strains as long as glucose was present in the environment, although vacuolar pH was different in the strains (Figure 2d and Figure S4b). In contrast, the *tor1∆*, *SIR2A*, and *tor1∆ SIR2A* strains, which exhibited decreased *PMA1* expression, showed lower pHc than that in the wild‐type, whereas *sir2∆*, *tor1∆ sir2∆*, and *tor1∆ SIR2E* strains, which exhibited increased *PMA1* expression, showed higher pHc than that in the wild‐type (Figures 2 and 3). Thus, it seems likely that the amount of Pma1 on the plasma membrane would be a major contributor to the change in pHc and vacuolar acidity in the *sir2∆* and *tor1∆* strains. These results are consistent with the previous observation that Pma1 accumulation on the plasma membrane is linked with reduced vacuolar acidity (Hughes & Gottschling, 2012).

A number of studies have reported that *TOR1* deletion or inhibition of TORC1 signaling by rapamycin or nutrient deprivation extends lifespan by sirtuin‐independent pathways in yeast (Kaeberlein et al., 2005; Powers, Kaeberlein, Caldwell, Kennedy, & Fields, 2006). However, there are also a few studies demonstrating that inhibition of TORC1 signaling enhances Sir2 activity to stabilize the rDNA loci and prevent the formation of ERCs, resulting in increased RLS in yeast (Ha & Huh, 2011; Medvedik et al., 2007). We demonstrated that TORC1 signaling regulates Sir2 phosphorylation (Figures 1d and 4f), which affects RLS through *PMA1* expression regulation independently of ERC formation (Kang et al., 2015). And, expression of the phospho‐mimetic *SIR2^S473E^* decreased RLS of the *tor1∆* mutant strain (Figure 3f), suggesting that part of the effect of *TOR1* deletion on RLS is related to Sir2 phosphorylation at S473. To more clearly show that Sir2 is placed downstream of TORC1 signaling in terms of the regulation of *PMA1* expression and RLS, we analyzed the effect of *SIR2* deletion on the RLS of *TOR1* or *KNS1* deletion strain. To exclude the effect of ERC formation on RLS, we deleted each gene in the *fob1∆* background and measured RLS. It was found that *SIR2* deletion reduced the RLS of *fob1∆ tor1∆* or *fob1∆ kns1∆* strain by 20.8% or 14.8%, respectively (Figure S8). Contrary to our expectation, however, the percent increase in RLS due to *TOR1* or *KNS1* deletion was greater in the absence of Sir2 (37.4% for *fob1∆ sir2∆ tor1∆* and 27.5% for *fob1∆ sir2∆ kns1∆*) than in the presence of Sir2 (32.9% for *fob1∆ tor1∆* and 16% for *fob1∆ kns1∆*), which is inconsistent with the Sir2’s role in promoting longevity. Interestingly, similar observations have been reported that the effect of *HXK2* or *GPA2* deletion on the percent extension in RLS was greater in the *fob1∆ sir2∆* background (67.2% or 53.8%, respectively) than in the *fob1∆* background (45.1% or 39%, respectively) (Delaney, Murakami, Olsen, Kennedy, & Kaeberlein, 2011; Kaeberlein, Kirkland, Fields, & Kennedy, 2004). These results suggest that the data of RLS analysis should be carefully interpreted to determine the epistasis relationship between two or more RLS‐altering genes. Especially with regard to *SIR2*, *TOR1*, and *KNS1*, which have multiple downstream effectors affecting cellular growth and RLS and form unknown and intricate interconnections between the downstream effectors (He, Zhou, & Kennedy, 2018), more sophisticatedly designed biochemical and genetic analyses are expected to reveal the relationship between TORC1 signaling and Sir2 in terms of RLS extension.

In summary, this study proposes a novel signaling network where two important nutrient signaling pathways, TORC1 and cAMP/PKA, converge on CK2, which regulates Sir2 phosphorylation to control *PMA1* expression and pHc in a glucose‐dependent manner. Considering that pHc acts as a cellular signal to control TORC1 and PKA activity in response to glucose availability (Dechant et al., 2014), we suggest that pHc and TORC1 and cAMP/PKA signaling pathways form a kind of positive feedback loop to regulate cell growth in response to glucose availability. Why this feedback loop is needed for growth control and how sophisticatedly it operates remain to be elucidated.

## 4 EXPERIMENTAL PROCEDURES

### 4.1 Yeast strains and growth conditions

The experiments were performed using the BY4741 (*MATa his3∆1 leu2∆0 met15∆0 ura3∆0*) strain unless otherwise noted. Cells were grown in standard YPD liquid media containing Bacto peptone 20 g/L (Becton Dickinson), yeast extract 10 g/L (Becton Dickinson), and dextrose 20 g/L (Junsei) and incubated at 30°C. Solid medium was prepared by adding 20 g/L agar (Junsei). Synthetic drop‐out medium was prepared by adding 0.67 g/L yeast nitrogen base without amino acids (Becton Dickinson) and amino acids, except for either uracil or histidine, to select yeast transformant cells. When required, transformants were plated onto solid medium containing 5′‐fluoroorotic acid (1 mg/ml) to select for the loss of the URA3 marker.

### 4.2 Molecular techniques

Deletion strains were generated by replacing each open reading frame with *URA3* or *HIS3* through homologous recombination, resulting in strains listed in Table S1 in the supplemental material. Disruption cassettes were amplified using the appropriate primers and plasmid templates listed in Table S2. Yeast cells were transformed using the lithium acetate method as described previously (Gietz & Schiestl, 2007), and successful transformants were selected on SC‐URA plates. To measure Pma1 protein levels by Western blotting, the endogenous *PMA1* gene was replaced with a DNA fragment containing the C‐terminal FLAG‐tagged *PMA1* and *URA3* marker genes by homologous recombination. To generate strains expressing Kns1 without catalytic activity (Kns1^D440A^), a DNA fragment including the FLAG‐tagged mutant *KNS1* and *URA3* (marker gene) was integrated at the endogenous *KNS1* region. All strains generated in this study were verified by PCR and/or Western blotting.

### 4.3 Stress resistance test

Yeast cells were grown in YPD medium at 30°C for overnight and then seeded into 2 ml of YPD medium at an initial OD600 of 0.2 and incubated to log phase. One milliliter of the cell culture was collected, and the cells were washed with distilled water, diluted, and spotted onto regular YPD or YPD containing 0.8 M NaCl. The cells were incubated for 2–3 days at 30°C, and then, the plates were photographed.

### 4.4 Preparation of whole‐cell extracts and Western blotting

All the strains were grown in YPD till the exponential phase at 30°C. Total cell extracts were prepared using the trichloroacetic acid (TCA) method, and the pellet was resuspended in sample mercaptoethanol, 0.001% bromophenol blue. Proteins were separated on 10% SDS–polyacrylamide gel electrophoresis (PAGE) gels and transferred to polyvinylidene fluoride (PVDF) membranes (Millipore). The membranes were probed with specific antibodies and detected by HRP‐conjugated secondary antibody. The primary antibodies were anti‐FLAG (1:1,000; Sigma) and anti‐GAPDH (1:20,000; Acris). Band density trace and quantification were determined using ImageJ (National Institutes of Health).

### 4.5 Immunoprecipitation

WT strains expressing chromosomally tagged KNS1‐13myc and SIT4‐HA was grown in YPD till the exponential phase at 30°C and then treated with or without 300 nM rapamycin for 1 hr and harvested cells were lysed in lysis buffer [50 mM NaCl, 150 mM Tris‐HCl pH 8.0, 0.1% NP40, 1 mM EDTA, 1 mM EGTA, 1 mM PMSF]. The supernatants were immunoprecipitated with anti‐HA (Millipore) antibody and followed by incubation with protein A/G beads (Santa Cruz Biotechnology). Immunoprecipitates were washed four times with lysis buffer and then eluted by boiling in sample buffer. Samples were resolved by 8% SDS‐PAGE and analyzed by Western blotting using the appropriate antibodies. To analyze the Sir2 phosphorylation level in the indicated strains, cells with chromosomally tagged SIR2‐13MYC were grown to the exponential phase at 30°C in YPD, harvested, and lysed in lysis buffer [50 mM HEPES‐KOH pH 7.5, 150 mM NaCl, 1 mM EDTA, 1% Triton X‐100, 1 mM PMSF] containing phosphatase inhibitors. Supernatants were collected and immunoprecipitated with anti‐Myc antibody (Millipore), and followed by incubation with 20 μl protein A/G beads (Santa Cruz Biotechnology). Samples were resolved by 8% SDS‐PAGE and analyzed by Western blotting using a phosphoserine antibody (1:100, Qiagen, Valencia, CA).

### 4.6 RNA isolation, cDNA synthesis, and real‐time PCR analysis

All the strains were grown in YPD till the exponential phase at 30°C. Total RNA was purified using the NucleoSpin RNA kit (Macherey‐Nagel) and quantified by measuring absorbance at 260 nm. From each 1 μg of RNA sample, cDNA was synthesized using the ReverTra Ace qPCR RT Kit (Toyobo, Japan), according to the manufacturer's recommendations, and analyzed by quantitative RT–PCR. RT–PCR was performed with SYBR green PCR mix (Bio‐Rad) and CFX connect system (Bio‐Rad). Relative expression levels (normalized to *ACT1*) were determined using the comparative CT method.

### 4.7 Yeast lifespan determination

The RLS of the yeast strains was determined by micromanipulation as previously described (Kaeberlein et al., 2005) using 60–70 virgin cells grown on standard YPD plates containing 2% glucose. Statistical significance of the difference in the RLS between strains was determined by Student's *t* test (*p* < .05).

### 4.8 Vacuole staining

Quinacrine (Sigma Q3251) staining was performed as previously described (Hughes & Gottschling, 2012) with some modification. Cells were grown in YPD media to an OD600 of 0.8–1.0, and 1 ml was harvested and washed twice in uptake buffer (YPD buffered to pH 7.6 with 100 mM HEPES). The cell pellet was resuspended in 100 µl of the same buffered media containing 200 μM quinacrine. Cells were incubated at 30℃ for 10 min and then for 5 min on ice. Cells were pelleted and washed twice with ice‐cold 100 mM HEPES (pH 7.6) and 2% glucose for imaging. Before imaging, cells were maintained on ice, and all images were obtained within 1 hr of staining.

### 4.9 Measurement of pHc

To generate pH calibration curve (Figure S3), yeast cells expressing SEP (a gift from Daniel E. Gottschling) were grown in baffled flasks to an OD600 of approximately 1.0 in YPD medium, centrifuged for 5 min at 850×*g*, washed two times with PBS, and resuspended in PBS containing 5 µg/ml digitonin (Sigma). After 5 min, the cells were washed with PBS and resuspended in citric acid/Na_2_HPO_4_ buffer, with pH values ranging from 5.0 to 8.5. Images were captured using an Olympus BX51 microscope (green fluorescent protein (GFP) excitation and emission, 460–490 and 520 nm, respectively). Image analysis was performed using ImageJ (version 1.43m, NIH) to quantify mean pHluorin intensity from three different regions of images with a 1‐pixel straight line tool. The background fluorescence of SEP‐nonproducing wild‐type cells was subtracted from those of SEP‐producing cells. Exponentially growing cells were used to quantify pHc of viable single cells. The fluorescence intensity was measured, plotted against the corresponding buffer pH, and fitted to the pH calibration curve (third‐order polynomial regression curve). pH values are always represented as mean ± *SD*. All pH determination experiments were repeated at least three times (biological replicates), and figures show one representative experiment in which error bars represent the standard deviation of at least three technical replicates.

### 4.10 V‐ATPase assembly assay

V‐ATPase assembly was investigated by co‐localization of Vph1‐mCherry (a gift from Daniel E. Gottschling) with Vma5‐Venus. All images were captured using an Olympus BX51 microscope. Cells co‐expressing Vph1‐mCherry and Vma5‐Venus were grown to the exponential phase in YEPD, and their intracellular localization was analyzed by fluorescence microscopy. Co‐localization was assessed in at least 30 cells from two to three independent experiments. Combined fluorescence intensity profile plots of Vph1‐mCherry and Vma5‐Venus were measured along the line displayed in each panel. The x‐axis indicated the distance along the line in pixels, whereas the y‐axis indicated the relative mCherry or Venus signal intensity. Pearson's coefficient was calculated using the ImageJ plugin JACoP. Results depicted are mean values ± 95% CI (one‐way ANOVA).

### 4.11 Image acquisition

Images were captured using an Olympus BX51 microscope (4′,6‐diamidino‐2‐phenylindole (DAPI) excitation and emission, 330–385 and 420 nm, respectively; GFP excitation and emission, 460–490 nm and 520 nm, respectively; red fluorescent protein (RFP) excitation and emission 530–550 and 575 nm, respectively).

## CONFLICT OF INTEREST

The authors declare no conflict of interest.

## AUTHOR CONTRIBUTIONS

MND conducted experiments and wrote the manuscript; YHK conducted experiments and wrote the manuscript; JJ and WKK conducted experiments; K‐SK conceived the concept, interpreted the results, and edited the manuscript; J‐YK conceived the concept, supervised the study, and wrote and edited the manuscript. All authors approved the manuscript.

## Supporting information

Supinfo

## Data Availability

The data that support the findings of this study are available from the corresponding author upon reasonable request.
