# Supplementary material for: TORC1 signaling regulates cytoplasmic pH through Sir2 in yeast
Source: Aging Cell. 2020 May 25;19(6):e13151. doi: 10.1111/acel.13151 (PMC7294778; doi:10.1111/acel.13151)
Supplement: Supplementary file 1 — Supinfo [file ACEL-19-e13151-s001.pptx]

## Slide 1
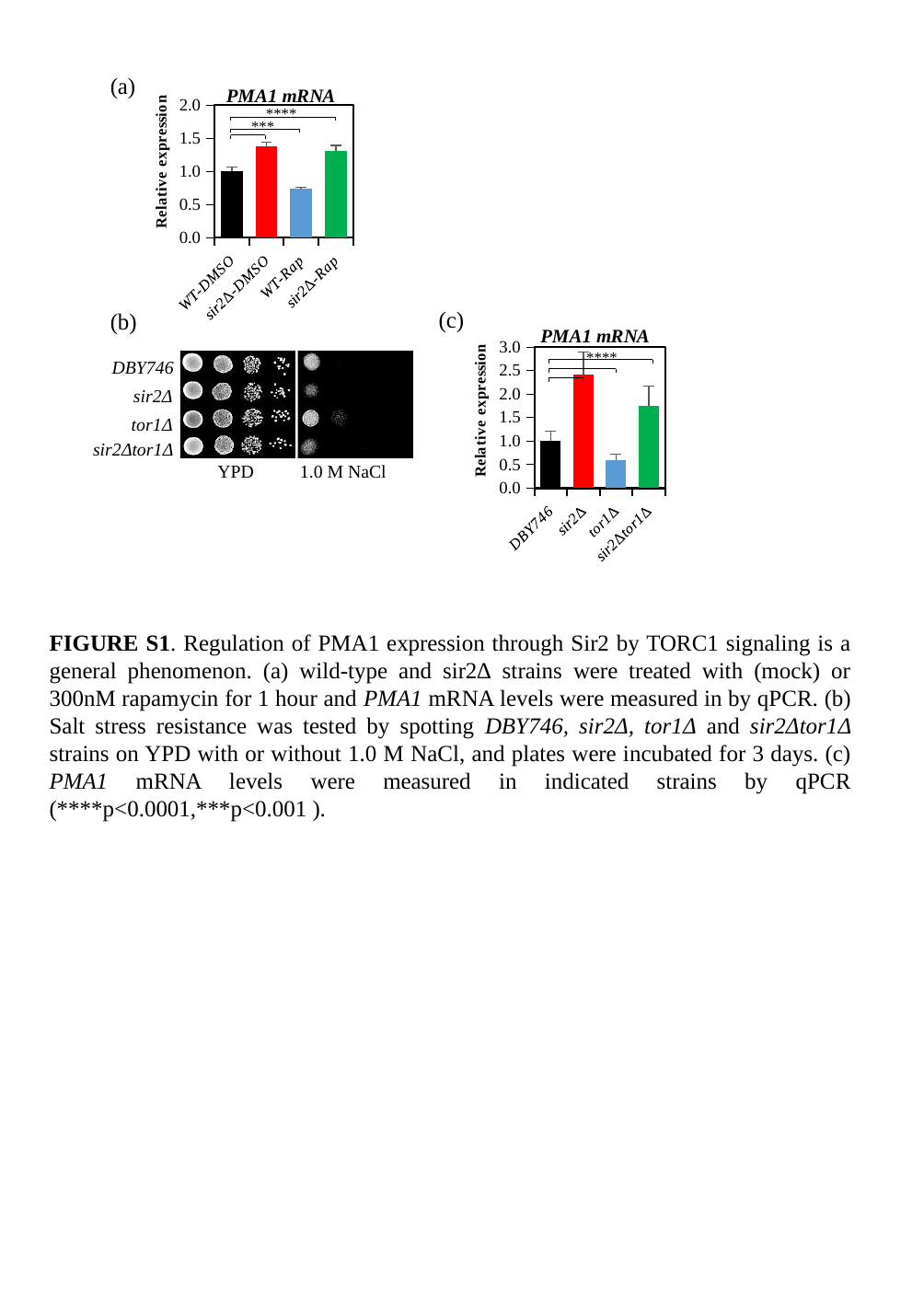

(a)
### Chart: PMA1 mRNA
| Category | |
|---|---|
| WT-DMSO | 1.002850847569495 |
| sir2∆-DMSO | 1.3841869556330926 |
| WT-Rap | 0.7363855609097114 |
| sir2∆-Rap | 1.3026173182399248 |****
***
(c)
(b)
### Chart: PMA1 mRNA
| Category | |
|---|---|
| DBY746 | 0.9973550238523763 |
| sir2∆ | 2.4068265454365005 |
| tor1∆ | 0.5899876492036361 |
| sir2∆tor1∆ | 1.7454028873975986 |****
DBY746
sir2Δtor1Δ
YPD 1.0 M NaCl
sir2Δ
tor1Δ
FIGURE S1. Regulation of PMA1 expression through Sir2 by TORC1 signaling is a general phenomenon. (a) wild-type and sir2∆ strains were treated with (mock) or 300nM rapamycin for 1 hour and PMA1 mRNA levels were measured in by qPCR. (b) Salt stress resistance was tested by spotting DBY746, sir2Δ, tor1Δ and sir2Δtor1Δ strains on YPD with or without 1.0 M NaCl, and plates were incubated for 3 days. (c) PMA1 mRNA levels were measured in indicated strains by qPCR (****p<0.0001,***p<0.001 ).

## Slide 2
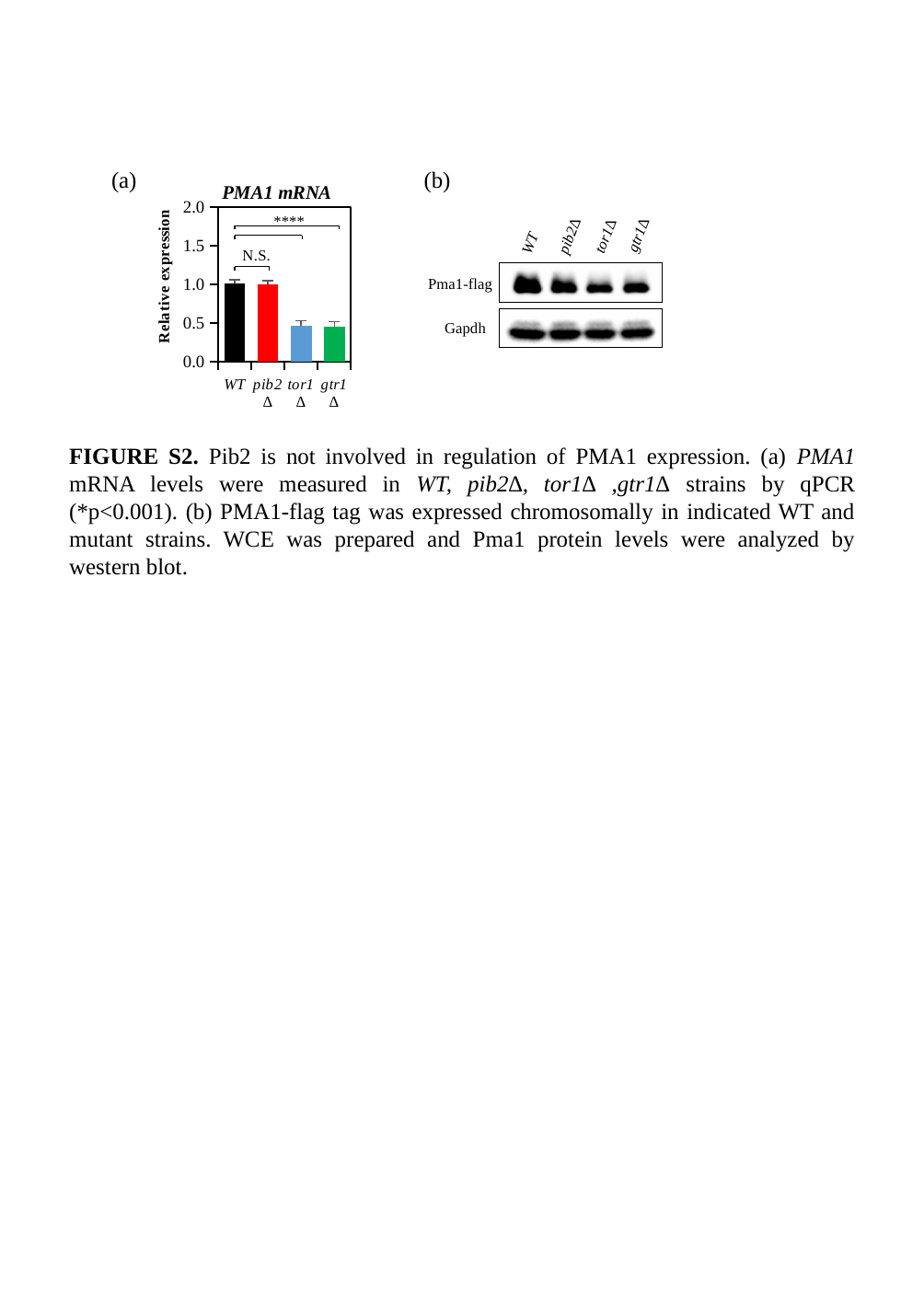

(a)
(b)
### Chart: PMA1 mRNA
| Category | |
|---|---|
| WT | 1.0153099398964853 |
| pib2∆ | 0.9970598953654086 |
| tor1∆ | 0.46431797454037116 |
| gtr1∆ | 0.45400257128467564 |****
gtr1∆
pib2∆
tor1∆
WT
Pma1-flag
Gapdh
FIGURE S2. Pib2 is not involved in regulation of PMA1 expression. (a) PMA1 mRNA levels were measured in WT, pib2∆, tor1∆ ,gtr1∆ strains by qPCR (*p<0.001). (b) PMA1-flag tag was expressed chromosomally in indicated WT and mutant strains. WCE was prepared and Pma1 protein levels were analyzed by western blot.

## Slide 3
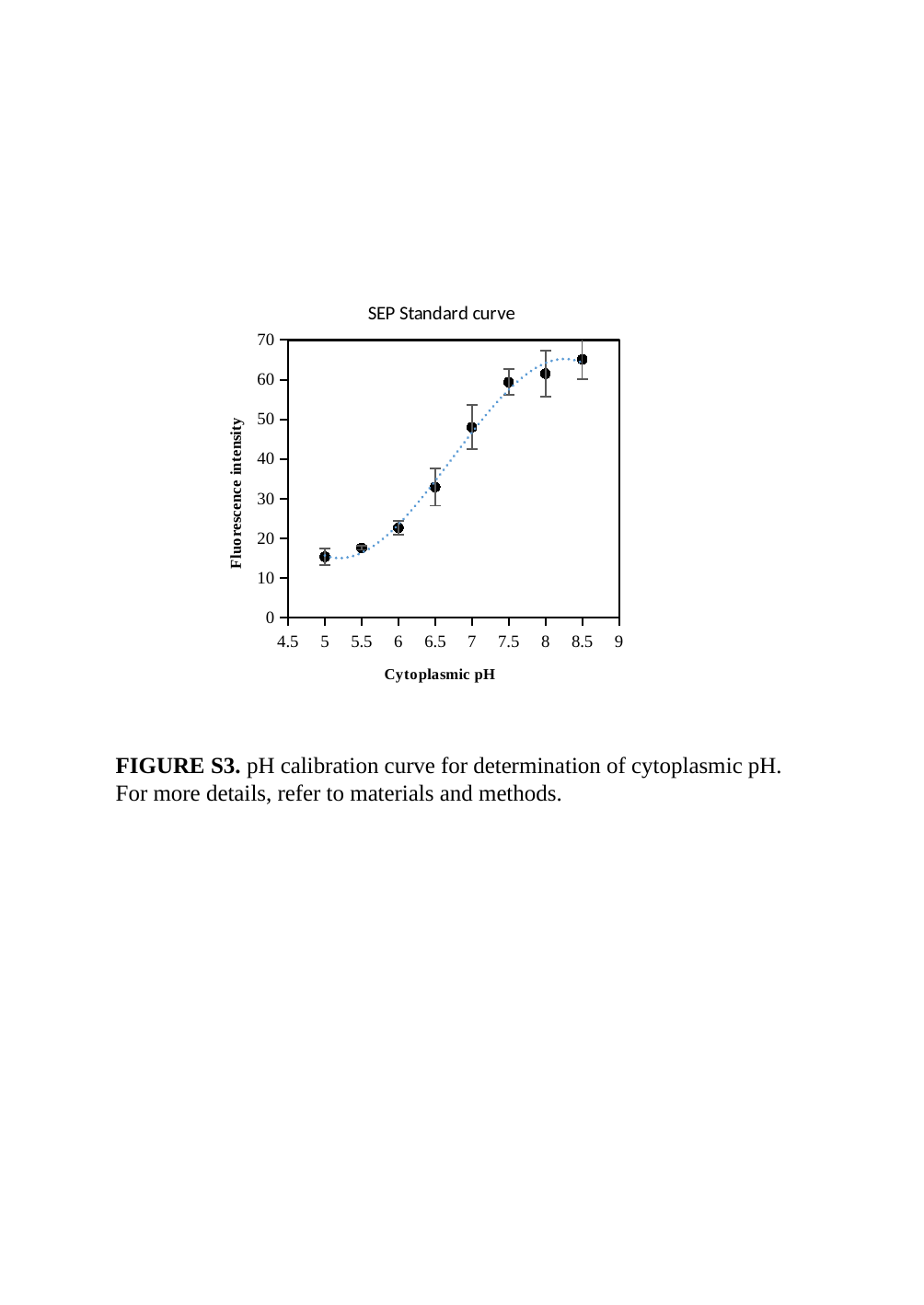

### Chart: SEP Standard curve
| Category | |
|---|---|FIGURE S3. pH calibration curve for determination of cytoplasmic pH.
For more details, refer to materials and methods.

## Slide 4
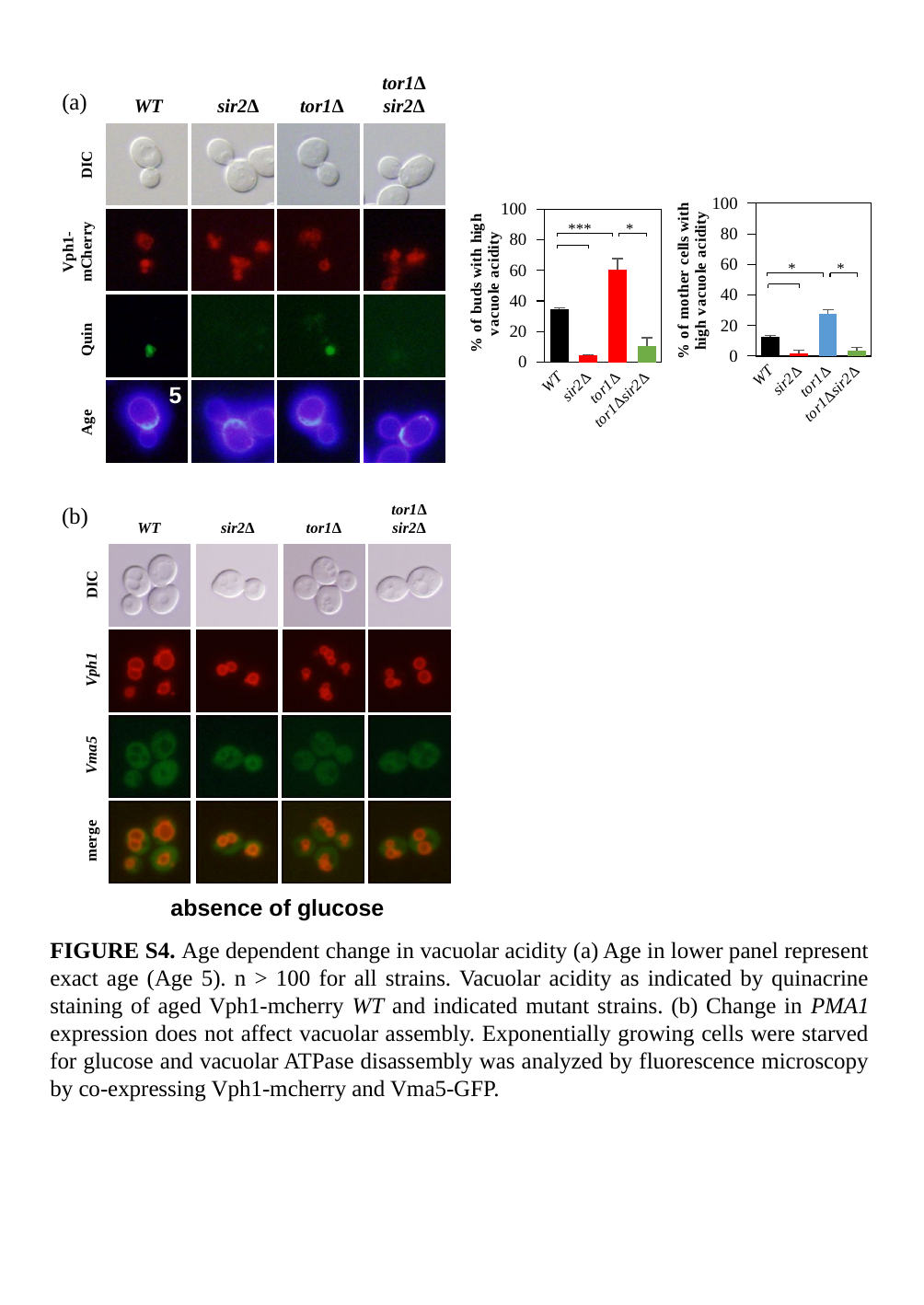

tor1∆
sir2∆
WT
sir2∆
tor1∆
DIC
Vph1-mCherry
Quin
5
Age
(a)
### Chart
| Category | |
|---|---|
| WT | 34.43024366937411 |
| sir2∆ | 4.819976771196283 |
| tor1∆ | 60.48805815160955 |
| tor1∆sir2∆ | 10.219780219780219 |***
*
### Chart
| Category | |
|---|---|
| WT | 12.571667462971812 |
| sir2∆ | 1.5873015873015872 |
| tor1∆ | 27.898926964347524 |
| tor1∆sir2∆ | 3.2902391725921136 |*
*
tor1∆
sir2∆
WT
sir2∆
tor1∆
absence of glucose
DIC
Vph1
Vma5
merge
(b)
FIGURE S4. Age dependent change in vacuolar acidity (a) Age in lower panel represent exact age (Age 5). n > 100 for all strains. Vacuolar acidity as indicated by quinacrine staining of aged Vph1-mcherry WT and indicated mutant strains. (b) Change in PMA1 expression does not affect vacuolar assembly. Exponentially growing cells were starved for glucose and vacuolar ATPase disassembly was analyzed by fluorescence microscopy by co-expressing Vph1-mcherry and Vma5-GFP.

## Slide 5
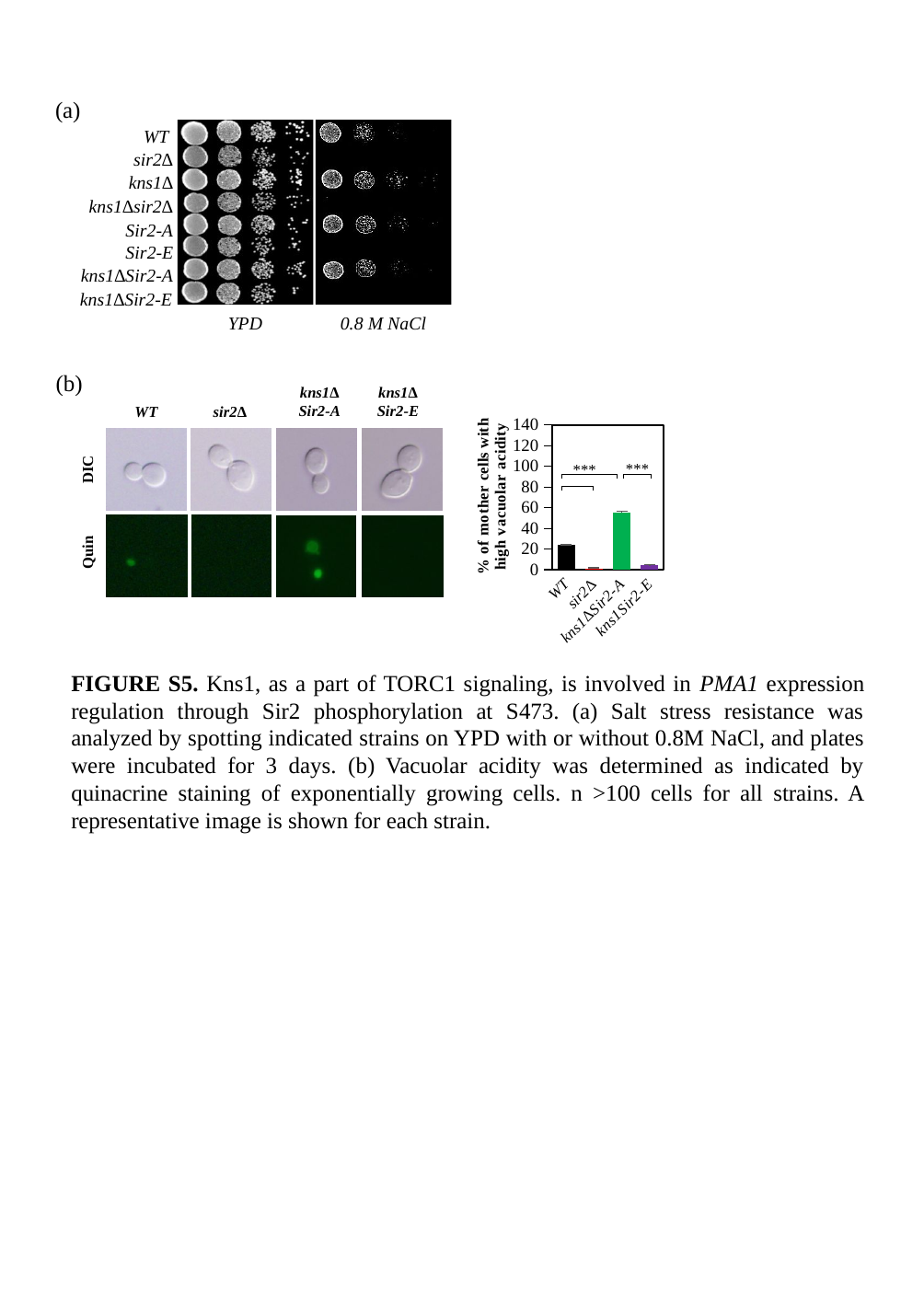

(a)
WT
sir2∆
kns1∆
kns1∆sir2∆
Sir2-A
Sir2-E
kns1∆Sir2-A
kns1∆Sir2-E
YPD
0.8 M NaCl
(b)
kns1∆
Sir2-E
kns1∆
Sir2-A
WT
sir2∆
DIC
Quin
### Chart
| Category | |
|---|---|
| WT | 23.70381556428068 |
| sir2∆ | 0.7142857142857143 |
| kns1∆Sir2-A | 55.423220973782776 |
| kns1Sir2-E | 4.628143392188337 |***
***
FIGURE S5. Kns1, as a part of TORC1 signaling, is involved in PMA1 expression regulation through Sir2 phosphorylation at S473. (a) Salt stress resistance was analyzed by spotting indicated strains on YPD with or without 0.8M NaCl, and plates were incubated for 3 days. (b) Vacuolar acidity was determined as indicated by quinacrine staining of exponentially growing cells. n >100 cells for all strains. A representative image is shown for each strain.

## Slide 6
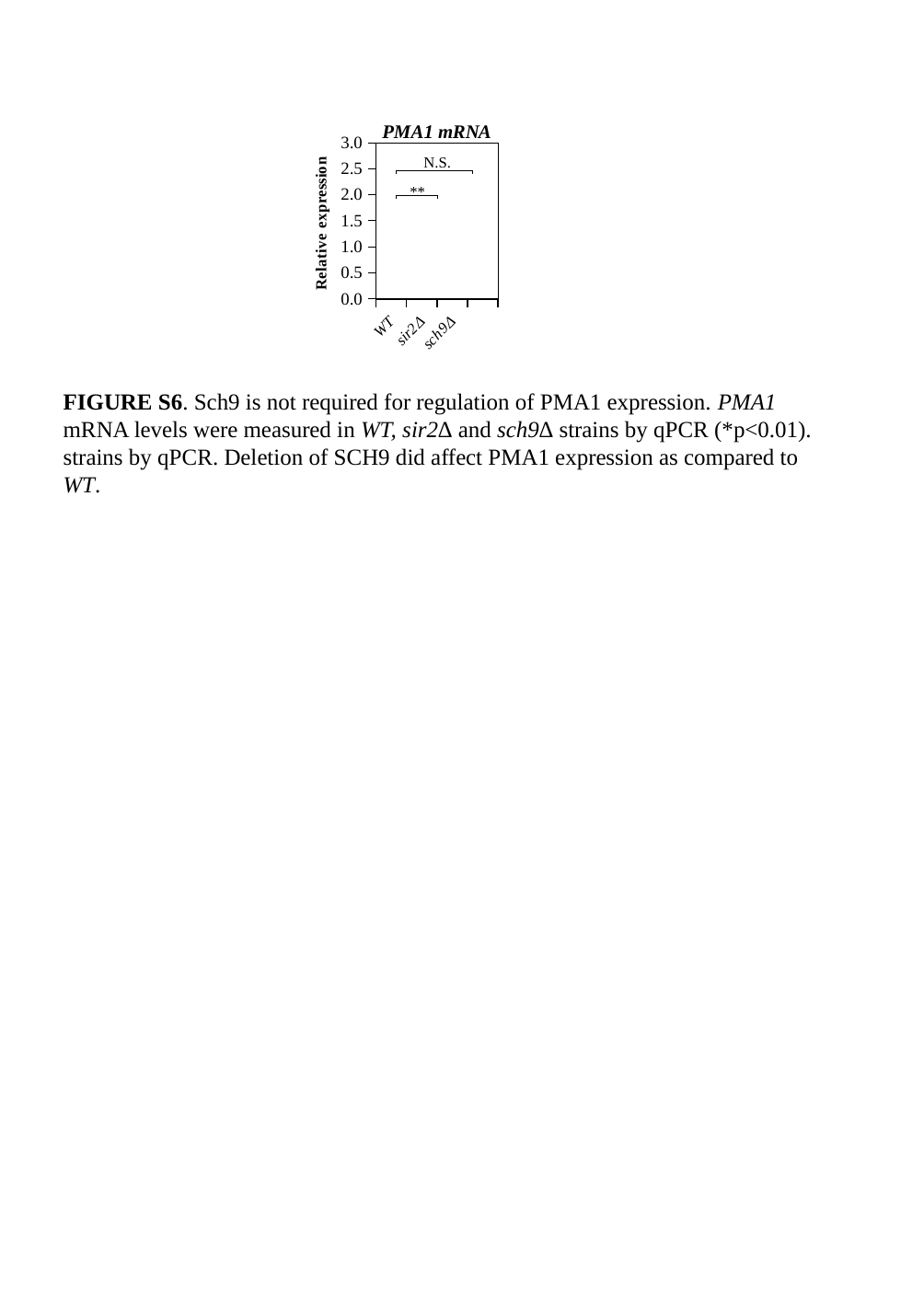

### Chart: PMA1 mRNA
| Category | PMA1 |
|---|---|
| WT | 0.9960574378836421 |
| sir2Δ | 1.6795298231050835 |
| sch9Δ | 0.9252245855373412 |N.S.
**
FIGURE S6. Sch9 is not required for regulation of PMA1 expression. PMA1 mRNA levels were measured in WT, sir2∆ and sch9∆ strains by qPCR (*p<0.01). strains by qPCR. Deletion of SCH9 did affect PMA1 expression as compared to WT.

## Slide 7
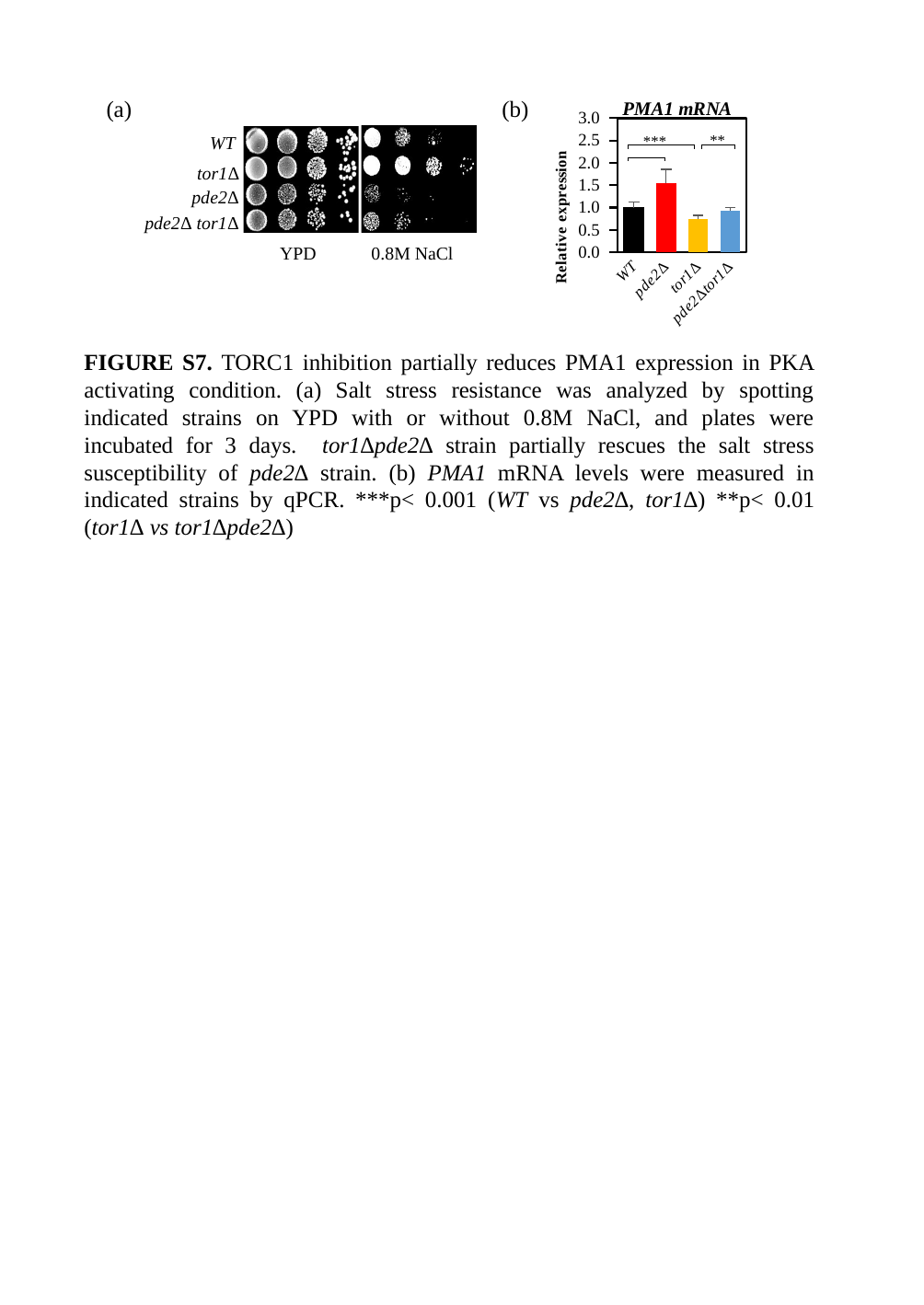

(b)
(a)
### Chart: PMA1 mRNA
| Category | |
|---|---|
| WT | 0.9988814264998602 |
| pde2∆ | 1.5445510113151524 |
| tor1∆ | 0.7330537529495067 |
| pde2∆tor1∆ | 0.9295317404584106 |**
***
WT
pde2Δ tor1Δ
 YPD 0.8M NaCl
tor1Δ
pde2Δ
FIGURE S7. TORC1 inhibition partially reduces PMA1 expression in PKA activating condition. (a) Salt stress resistance was analyzed by spotting indicated strains on YPD with or without 0.8M NaCl, and plates were incubated for 3 days. tor1∆pde2∆ strain partially rescues the salt stress susceptibility of pde2∆ strain. (b) PMA1 mRNA levels were measured in indicated strains by qPCR. ***p< 0.001 (WT vs pde2∆, tor1∆) **p< 0.01 (tor1∆ vs tor1∆pde2∆)

## Slide 8
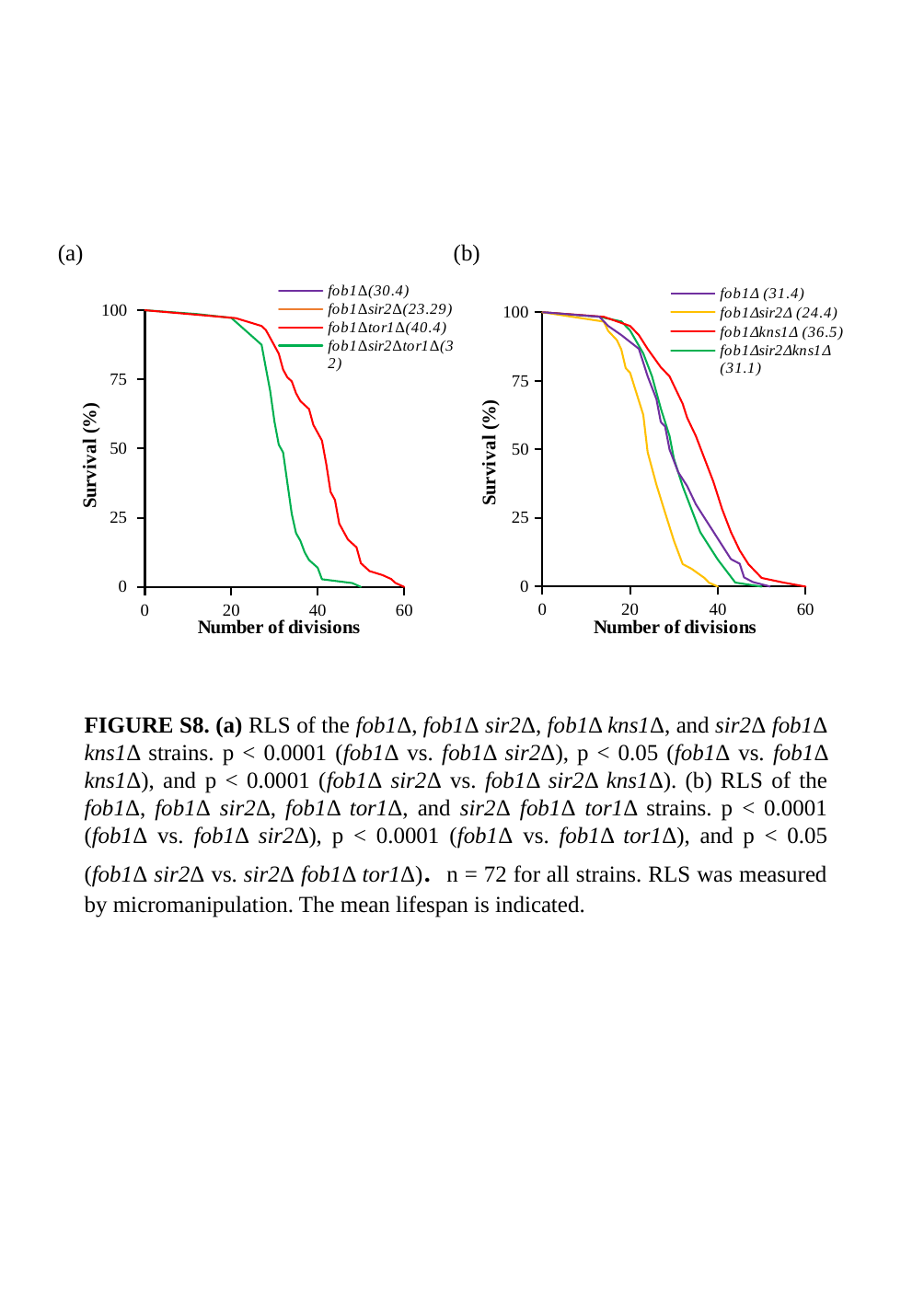

(a)
(b)
### Chart
| Category | fob1Δ (31.4) | fob1Δsir2Δ (24.4) | fob1Δkns1Δ (36.5) | fob1Δsir2Δkns1Δ (31.1) |
|---|---|---|---|---|
### Chart
| Category | fob1∆(30.4) | fob1∆sir2∆(23.29) | fob1∆tor1∆(40.4) | fob1∆sir2∆tor1∆(32) |
|---|---|---|---|---|FIGURE S8. (a) RLS of the fob1Δ, fob1Δ sir2Δ, fob1Δ kns1Δ, and sir2Δ fob1Δ kns1Δ strains. p < 0.0001 (fob1Δ vs. fob1Δ sir2Δ), p < 0.05 (fob1Δ vs. fob1Δ kns1Δ), and p < 0.0001 (fob1Δ sir2Δ vs. fob1Δ sir2Δ kns1Δ). (b) RLS of the fob1Δ, fob1Δ sir2Δ, fob1Δ tor1Δ, and sir2Δ fob1Δ tor1Δ strains. p < 0.0001 (fob1Δ vs. fob1Δ sir2Δ), p < 0.0001 (fob1Δ vs. fob1Δ tor1Δ), and p < 0.05 (fob1Δ sir2Δ vs. sir2Δ fob1Δ tor1Δ). n = 72 for all strains. RLS was measured by micromanipulation. The mean lifespan is indicated.
